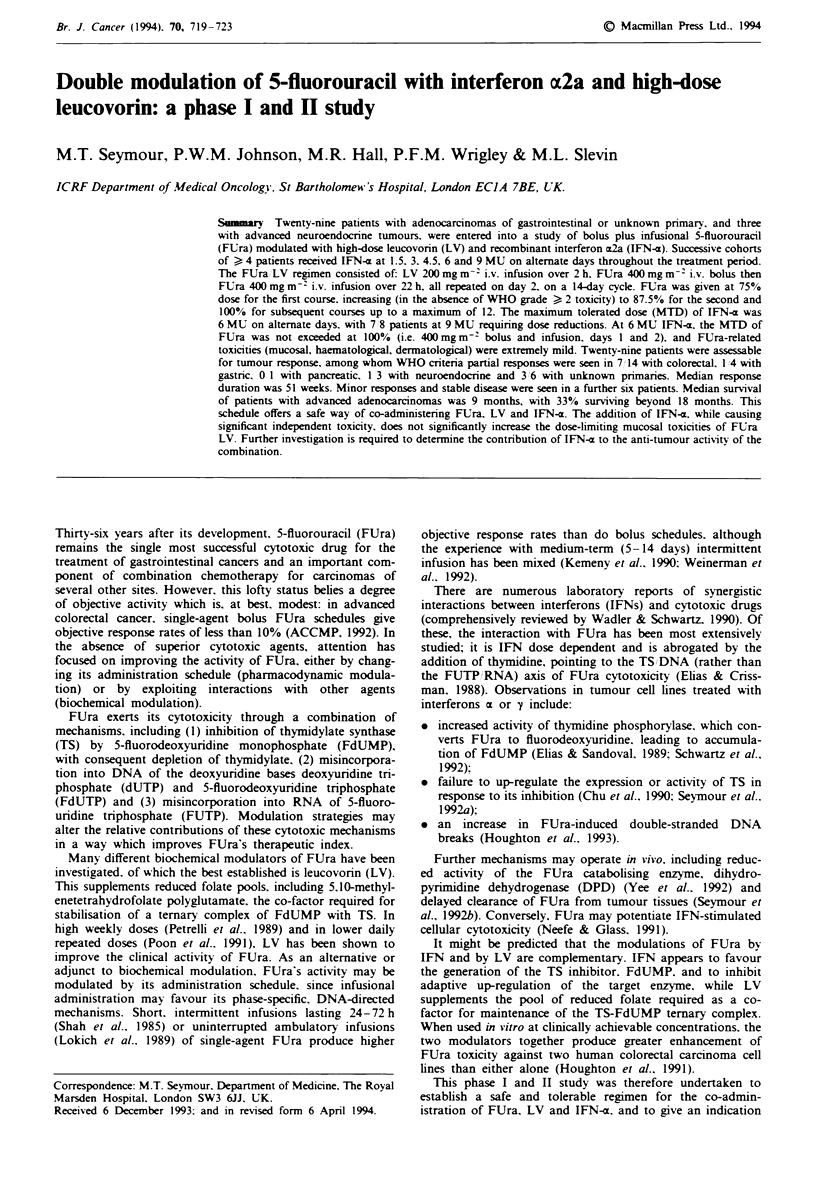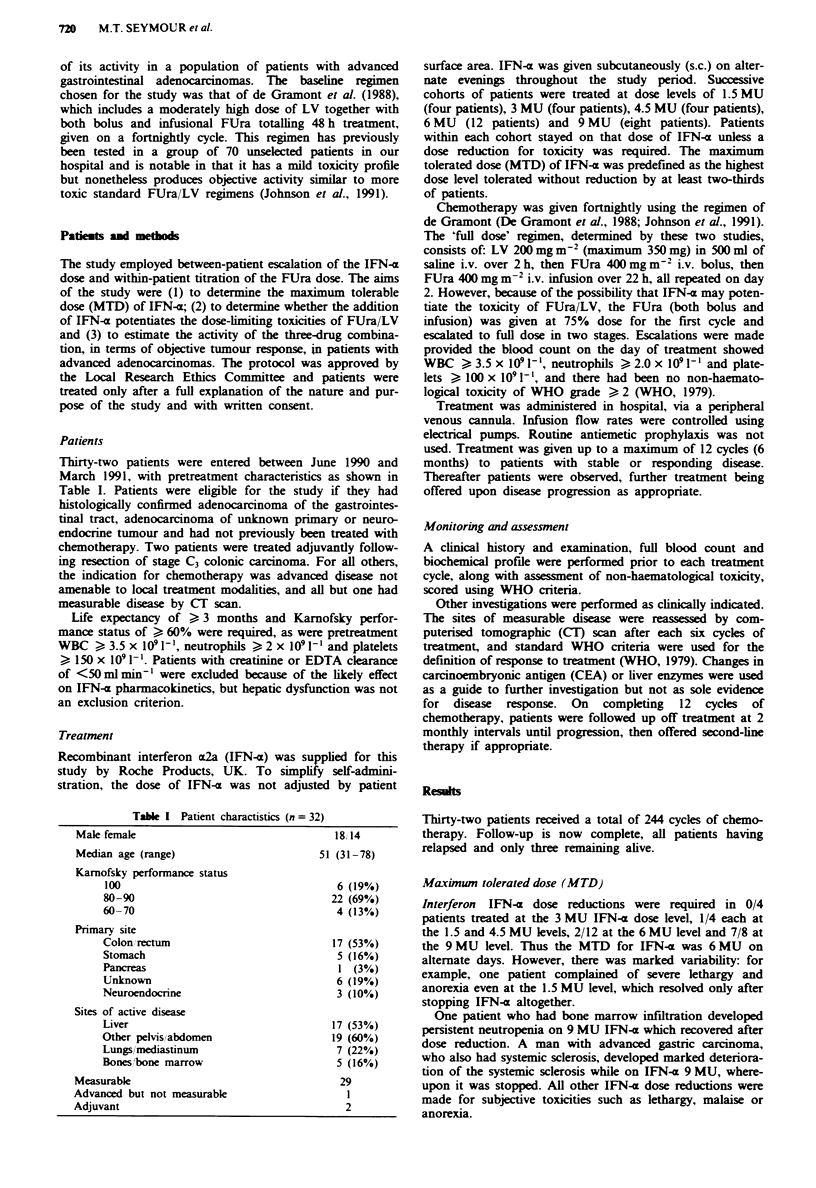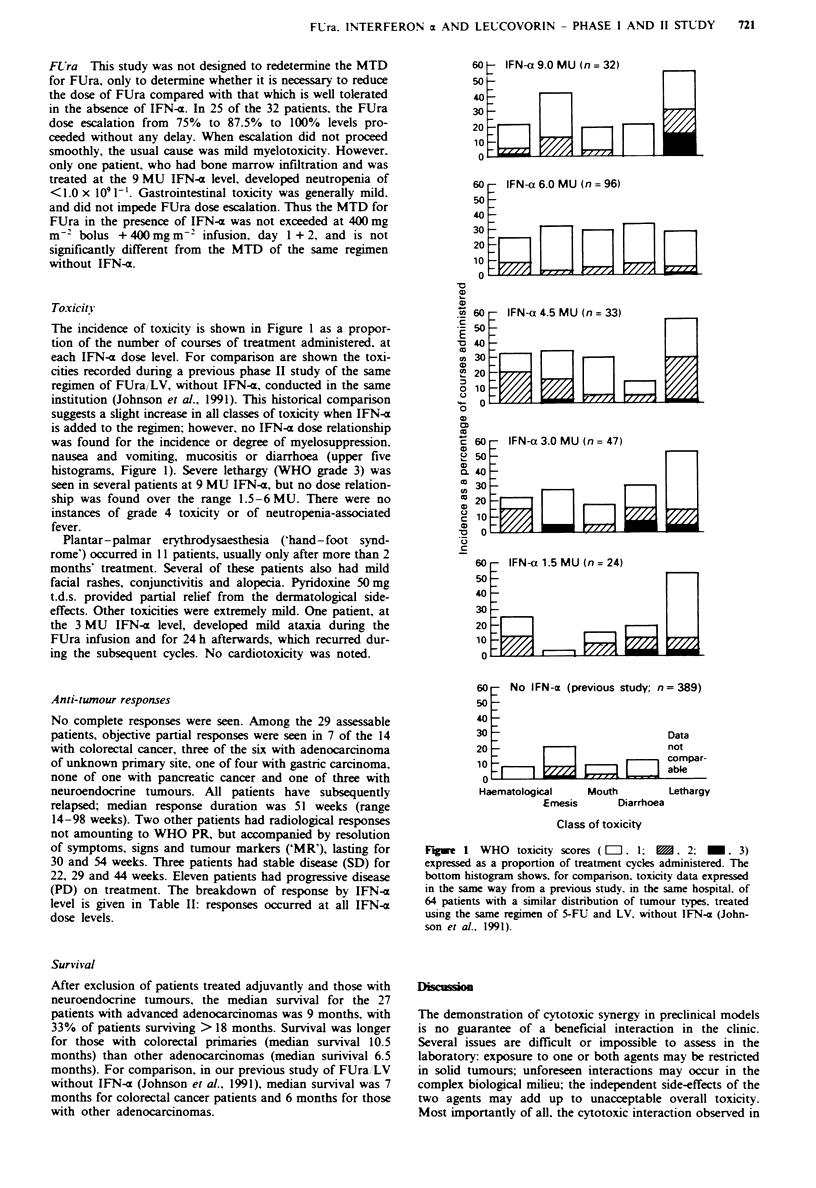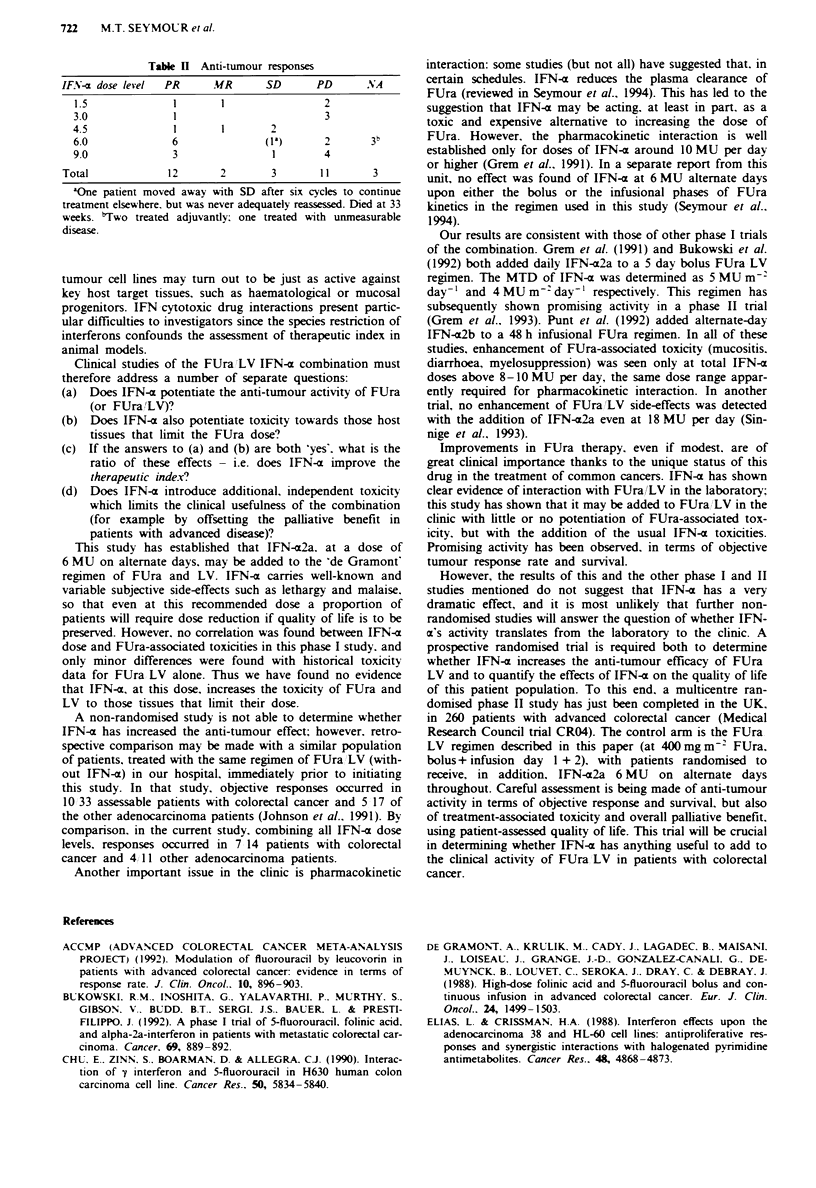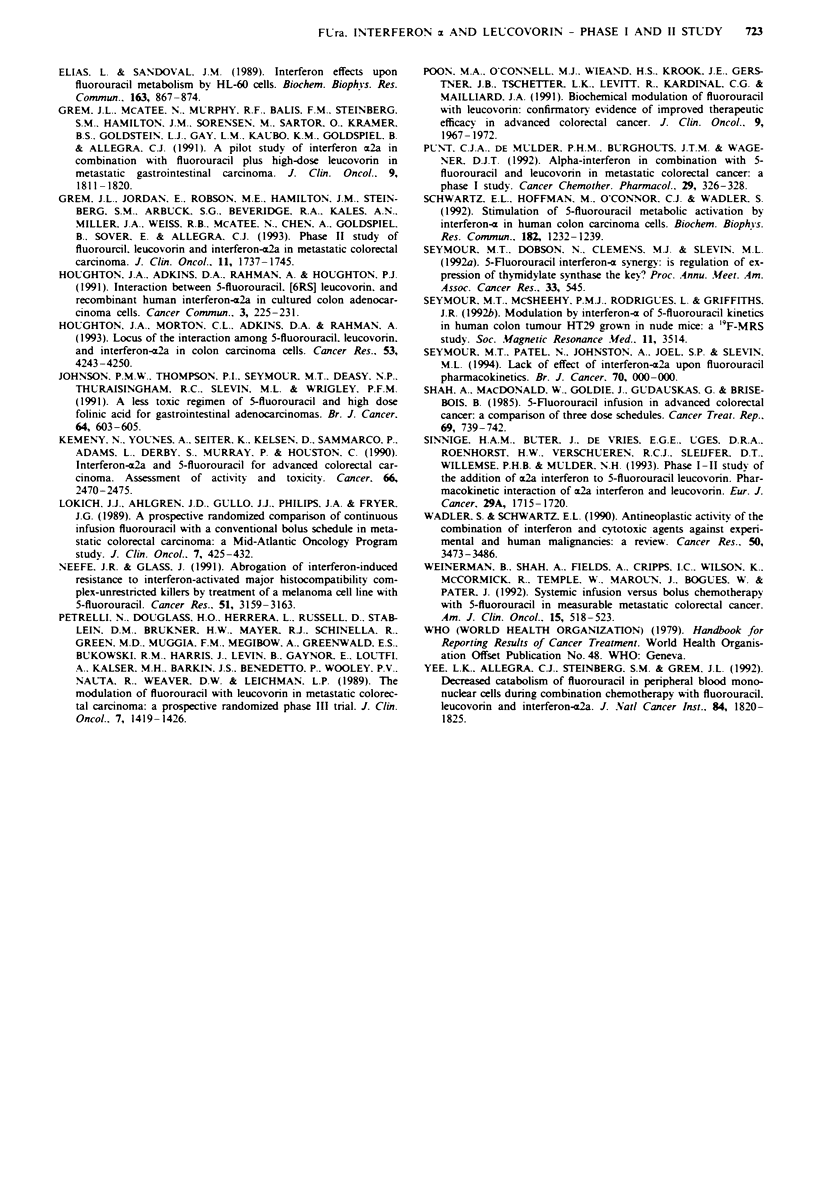# Double modulation of 5-fluorouracil with interferon alpha 2a and high-dose leucovorin: a phase I and II study.

**DOI:** 10.1038/bjc.1994.382

**Published:** 1994-10

**Authors:** M. T. Seymour, P. W. Johnson, M. R. Hall, P. F. Wrigley, M. L. Slevin

**Affiliations:** ICRF Department of Medical Oncology, St Bartholomew's Hospital, London, UK.

## Abstract

Twenty-nine patients with adenocarcinomas of gastrointestinal or unknown primary, and three with advanced neuroendocrine tumours, were entered into a study of bolus plus infusional 5-fluorouracil (FUra) modulated with high-dose leucovorin (LV) and recombinant interferon alpha 2a (IFN-alpha). Successive cohorts of > or = 4 patients received IFN-alpha at 1.5, 3, 4.5, 6 and 9 MU on alternate days throughout the treatment period. The FUra/LV regimen consisted of: LV 200 mg m-2 i.v. infusion over 2 h, FUra 400 mg m-2 i.v. bolus then FUra 400 mg m-2 i.v. infusion over 22 h, all repeated on day 2, on a 14-day cycle. FUra was given at 75% dose for the first course, increasing (in the absence of WHO grade > or = 2 toxicity) to 87.5% for the second and 100% for subsequent courses up to a maximum of 12. The maximum tolerated dose (MTD) of IFN-alpha was 6 MU on alternate days, with 7/8 patients at 9 MU requiring dose reductions. At 6 MU IFN-alpha, the MTD of FUra was not exceeded at 100% (i.e. 400 mg m-2 bolus and infusion, days 1 and 2), and FUra-related toxicities (mucosal, haematological, dermatological) were extremely mild. Twenty-nine patients were assessable for tumour response, among whom WHO criteria partial responses were seen in 7/14 with colorectal, 1/4 with gastric, 0/1 with pancreatic, 1/3 with neuroendocrine and 3/6 with unknown primaries. Median response duration was 51 weeks. Minor responses and stable disease were seen in a further six patients. Median survival of patients with advanced adenocarcinomas was 9 months, with 33% surviving beyond 18 months. This schedule offers a safe way of co-administering FUra, LV and IFN-alpha. The addition of IFN-alpha, while causing significant independent toxicity, does not significantly increase the dose-limiting mucosal toxicities of FUra/LV. Further investigation is required to determine the contribution of IFN-alpha to the anti-tumour activity of the combination.


Br. J. Cancer (1994), 70, 719–723 © Macmillan Press Ltd., 1994

### Double modulation of 5-fluorouracil with interferon α2a and high-dose leucovorin: a phase I and II study

M.T. Seymour, P.W.M. Johnson, M.R. Hall, P.F.M. Wrigley & M.L. Slevin

*ICRF Department of Medical Oncology, St Bartholomew's Hospital, London EC1A 7BE, UK.*

**Summary** Twenty-nine patients with adenocarcinomas of gastrointestinal or unknown primary, and three with advanced neuroendocrine tumours, were entered into a study of bolus plus infusional 5-fluorouracil (FUra) modulated with high-dose leucovorin (LV) and recombinant interferon α2a (IFN-α). Successive cohorts of ≥ 4 patients received IFN-α at 1.5, 3, 4.5, 6 and 9 MU on alternate days throughout the treatment period. The FUra/LV regimen consisted of: LV 200 mg m$^{-2}$ i.v. infusion over 2 h, FUra 400 mg m$^{-2}$ i.v. bolus then FUra 400 mg m$^{-2}$ i.v. infusion over 22 h, all repeated on day 2, on a 14-day cycle. FUra was given at 75% dose for the first course, increasing (in the absence of WHO grade ≥ 2 toxicity) to 87.5% for the second and 100% for subsequent courses up to a maximum of 12. The maximum tolerated dose (MTD) of IFN-α was 6 MU on alternate days, with 7/8 patients at 9 MU requiring dose reductions. At 6 MU IFN-α, the MTD of FUra was not exceeded at 100% (i.e. 400 mg m$^{-2}$ bolus and infusion, days 1 and 2), and FUra-related toxicities (mucosal, haematological, dermatological) were extremely mild. Twenty-nine patients were assessable for tumour response, among whom WHO criteria partial responses were seen in 7/14 with colorectal, 1/4 with gastric, 0/1 with pancreatic, 1/3 with neuroendocrine and 3/6 with unknown primaries. Median response duration was 51 weeks. Minor responses and stable disease were seen in a further six patients. Median survival of patients with advanced adenocarcinomas was 9 months, with 33% surviving beyond 18 months. This schedule offers a safe way of co-administering FUra, LV and IFN-α. The addition of IFN-α, while causing significant independent toxicity, does not significantly increase the dose-limiting mucosal toxicities of FUra/LV. Further investigation is required to determine the contribution of IFN-α to the anti-tumour activity of the combination.

Thirty-six years after its development, 5-fluorouracil (FUra) remains the single most successful cytotoxic drug for the treatment of gastrointestinal cancers and an important component of combination chemotherapy for carcinomas of several other sites. However, this lofty status belies a degree of objective activity which is, at best, modest: in advanced colorectal cancer, single-agent bolus FUra schedules give objective response rates of less than 10% (ACCMP, 1992). In the absence of superior cytotoxic agents, attention has focused on improving the activity of FUra, either by changing its administration schedule (pharmacodynamic modulation) or by exploiting interactions with other agents (biochemical modulation).

FUra exerts its cytotoxicity through a combination of mechanisms, including (1) inhibition of thymidylate synthase (TS) by 5-fluorodeoxyuridine monophosphate (FdUMP), with consequent depletion of thymidylate, (2) misincorporation into DNA of the deoxyuridine bases deoxyuridine triphosphate (dUTP) and 5-fluorodeoxyuridine triphosphate (FdUTP) and (3) misincorporation into RNA of 5-fluorouridine triphosphate (FUTP). Modulation strategies may alter the relative contributions of these cytotoxic mechanisms in a way which improves FUra's therapeutic index.

Many different biochemical modulators of FUra have been investigated, of which the best established is leucovorin (LV). This supplements reduced folate pools, including 5,10-methylenetetrahydrofolate polyglutamate, the co-factor required for stabilisation of a ternary complex of FdUMP with TS. In high weekly doses (Petrelli *et al.*, 1989) and in lower daily repeated doses (Poon *et al.*, 1991), LV has been shown to improve the clinical activity of FUra. As an alternative or adjunct to biochemical modulation, FUra's activity may be modulated by its administration schedule, since infusional administration may favour its phase-specific, DNA-directed mechanisms. Short, intermittent infusions lasting 24–72 h (Shah *et al.*, 1985) or uninterrupted ambulatory infusions (Lokich *et al.*, 1989) of single-agent FUra produce higher objective response rates than do bolus schedules, although the experience with medium-term (5–14 days) intermittent infusion has been mixed (Kemeny *et al.*, 1990; Weinerman *et al.*, 1992).

There are numerous laboratory reports of synergistic interactions between interferons (IFNs) and cytotoxic drugs (comprehensively reviewed by Wadler & Schwartz, 1990). Of these, the interaction with FUra has been most extensively studied; it is IFN dose dependent and is abrogated by the addition of thymidine, pointing to the TS/DNA (rather than the FUTP/RNA) axis of FUra cytotoxicity (Elias & Crissman, 1988). Observations in tumour cell lines treated with interferons α or γ include:

- increased activity of thymidine phosphorylase, which converts FUra to fluorodeoxyuridine, leading to accumulation of FdUMP (Elias & Sandoval, 1989; Schwartz *et al.*, 1992);
- failure to up-regulate the expression or activity of TS in response to its inhibition (Chu *et al.*, 1990; Seymour *et al.*, 1992*a*);
- an increase in FUra-induced double-stranded DNA breaks (Houghton *et al.*, 1993).

Further mechanisms may operate *in vivo*, including reduced activity of the FUra catabolising enzyme, dihydropyrimidine dehydrogenase (DPD) (Yee *et al.*, 1992) and delayed clearance of FUra from tumour tissues (Seymour *et al.*, 1992*b*). Conversely, FUra may potentiate IFN-stimulated cellular cytotoxicity (Neefe & Glass, 1991).

It might be predicted that the modulations of FUra by IFN and by LV are complementary. IFN appears to favour the generation of the TS inhibitor, FdUMP, and to inhibit adaptive up-regulation of the target enzyme, while LV supplements the pool of reduced folate required as a cofactor for maintenance of the TS-FdUMP ternary complex. When used *in vitro* at clinically achievable concentrations, the two modulators together produce greater enhancement of FUra toxicity against two human colorectal carcinoma cell lines than either alone (Houghton *et al.*, 1991).

This phase I and II study was therefore undertaken to establish a safe and tolerable regimen for the co-administration of FUra, LV and IFN-α, and to give an indication

Correspondence: M.T. Seymour, Department of Medicine, The Royal Marsden Hospital, London SW3 6JJ, UK.
Received 6 December 1993; and in revised form 6 April 1994.

of its activity in a population of patients with advanced gastrointestinal adenocarcinomas. The baseline regimen chosen for the study was that of de Gramont *et al.* (1988), which includes a moderately high dose of LV together with both bolus and infusional FUra totalling 48 h treatment, given on a fortnightly cycle. This regimen has previously been tested in a group of 70 unselected patients in our hospital and is notable in that it has a mild toxicity profile but nonetheless produces objective activity similar to more toxic standard FUra/LV regimens (Johnson *et al.*, 1991).

### Patients and methods

The study employed between-patient escalation of the IFN-α dose and within-patient titration of the FUra dose. The aims of the study were (1) to determine the maximum tolerable dose (MTD) of IFN-α; (2) to determine whether the addition of IFN-α potentiates the dose-limiting toxicities of FUra/LV and (3) to estimate the activity of the three-drug combination, in terms of objective tumour response, in patients with advanced adenocarcinomas. The protocol was approved by the Local Research Ethics Committee and patients were treated only after a full explanation of the nature and purpose of the study and with written consent.

### *Patients*

Thirty-two patients were entered between June 1990 and March 1991, with pretreatment characteristics as shown in Table I. Patients were eligible for the study if they had histologically confirmed adenocarcinoma of the gastrointestinal tract, adenocarcinoma of unknown primary or neuroendocrine tumour and had not previously been treated with chemotherapy. Two patients were treated adjuvantly following resection of stage $C_3$ colonic carcinoma. For all others, the indication for chemotherapy was advanced disease not amenable to local treatment modalities, and all but one had measurable disease by CT scan.

Life expectancy of ⩾3 months and Karnofsky performance status of ⩾60% were required, as were pretreatment WBC $\geqslant 3.5 \times 10^9\,l^{-1}$, neutrophils $\geqslant 2 \times 10^9\,l^{-1}$ and platelets $\geqslant 150 \times 10^9\,l^{-1}$. Patients with creatinine or EDTA clearance of $<50\,ml\,min^{-1}$ were excluded because of the likely effect on IFN-α pharmacokinetics, but hepatic dysfunction was not an exclusion criterion.

### *Treatment*

Recombinant interferon α2a (IFN-α) was supplied for this study by Roche Products, UK. To simplify self-administration, the dose of IFN-α was not adjusted by patient surface area. IFN-α was given subcutaneously (s.c.) on alternate evenings throughout the study period. Successive cohorts of patients were treated at dose levels of 1.5 MU (four patients), 3 MU (four patients), 4.5 MU (four patients), 6 MU (12 patients) and 9 MU (eight patients). Patients within each cohort stayed on that dose of IFN-α unless a dose reduction for toxicity was required. The maximum tolerated dose (MTD) of IFN-α was predefined as the highest dose level tolerated without reduction by at least two-thirds of patients.

Chemotherapy was given fortnightly using the regimen of de Gramont (De Gramont *et al.*, 1988; Johnson *et al.*, 1991). The 'full dose' regimen, determined by these two studies, consists of: LV $200\,mg\,m^{-2}$ (maximum 350 mg) in 500 ml of saline i.v. over 2 h, then FUra $400\,mg\,m^{-2}$ i.v. bolus, then FUra $400\,mg\,m^{-2}$ i.v. infusion over 22 h, all repeated on day 2. However, because of the possibility that IFN-α may potentiate the toxicity of FUra/LV, the FUra (both bolus and infusion) was given at 75% dose for the first cycle and escalated to full dose in two stages. Escalations were made provided the blood count on the day of treatment showed WBC $\geqslant 3.5 \times 10^9\,l^{-1}$, neutrophils $\geqslant 2.0 \times 10^9\,l^{-1}$ and platelets $\geqslant 100 \times 10^9\,l^{-1}$, and there had been no non-haematological toxicity of WHO grade ⩾2 (WHO, 1979).

Treatment was administered in hospital, via a peripheral venous cannula. Infusion flow rates were controlled using electrical pumps. Routine antiemetic prophylaxis was not used. Treatment was given up to a maximum of 12 cycles (6 months) to patients with stable or responding disease. Thereafter patients were observed, further treatment being offered upon disease progression as appropriate.

### *Monitoring and assessment*

A clinical history and examination, full blood count and biochemical profile were performed prior to each treatment cycle, along with assessment of non-haematological toxicity, scored using WHO criteria.

Other investigations were performed as clinically indicated. The sites of measurable disease were reassessed by computerised tomographic (CT) scan after each six cycles of treatment, and standard WHO criteria were used for the definition of response to treatment (WHO, 1979). Changes in carcinoembryonic antigen (CEA) or liver enzymes were used as a guide to further investigation but not as sole evidence for disease response. On completing 12 cycles of chemotherapy, patients were followed up off treatment at 2 monthly intervals until progression, then offered second-line therapy if appropriate.

### Results

Thirty-two patients received a total of 244 cycles of chemotherapy. Follow-up is now complete, all patients having relapsed and only three remaining alive.

### *Maximum tolerated dose (MTD)*

*Interferon* IFN-α dose reductions were required in 0/4 patients treated at the 3 MU IFN-α dose level, 1/4 each at the 1.5 and 4.5 MU levels, 2/12 at the 6 MU level and 7/8 at the 9 MU level. Thus the MTD for IFN-α was 6 MU on alternate days. However, there was marked variability: for example, one patient complained of severe lethargy and anorexia even at the 1.5 MU level, which resolved only after stopping IFN-α altogether.

One patient who had bone marrow infiltration developed persistent neutropenia on 9 MU IFN-α which recovered after dose reduction. A man with advanced gastric carcinoma, who also had systemic sclerosis, developed marked deterioration of the systemic sclerosis while on IFN-α 9 MU, whereupon it was stopped. All other IFN-α dose reductions were made for subjective toxicities such as lethargy, malaise or anorexia.

**Table I** Patient charactistics ($n = 32$)

| | |
|---|---|
| Male/female | 18/14 |
| Median age (range) | 51 (31–78) |
| Karnofsky performance status | |
| 100 | 6 (19%) |
| 80–90 | 22 (69%) |
| 60–70 | 4 (13%) |
| Primary site | |
| Colon/rectum | 17 (53%) |
| Stomach | 5 (16%) |
| Pancreas | 1 (3%) |
| Unknown | 6 (19%) |
| Neuroendocrine | 3 (10%) |
| Sites of active disease | |
| Liver | 17 (53%) |
| Other pelvis/abdomen | 19 (60%) |
| Lungs/mediastinum | 7 (22%) |
| Bones/bone marrow | 5 (16%) |
| Measurable | 29 |
| Advanced but not measurable | 1 |
| Adjuvant | 2 |

*FUra* This study was not designed to redetermine the MTD for FUra, only to determine whether it is necessary to reduce the dose of FUra compared with that which is well tolerated in the absence of IFN-α. In 25 of the 32 patients, the FUra dose escalation from 75% to 87.5% to 100% levels proceeded without any delay. When escalation did not proceed smoothly, the usual cause was mild myelotoxicity. However, only one patient, who had bone marrow infiltration and was treated at the 9 MU IFN-α level, developed neutropenia of $<1.0 \times 10^9\,l^{-1}$. Gastrointestinal toxicity was generally mild, and did not impede FUra dose escalation. Thus the MTD for FUra in the presence of IFN-α was not exceeded at 400 mg $m^{-2}$ bolus + 400 mg $m^{-2}$ infusion, day 1 + 2, and is not significantly different from the MTD of the same regimen without IFN-α.

### *Toxicity*

The incidence of toxicity is shown in Figure 1 as a proportion of the number of courses of treatment administered, at each IFN-α dose level. For comparison are shown the toxicities recorded during a previous phase II study of the same regimen of FUra/LV, without IFN-α, conducted in the same institution (Johnson *et al.*, 1991). This historical comparison suggests a slight increase in all classes of toxicity when IFN-α is added to the regimen; however, no IFN-α dose relationship was found for the incidence or degree of myelosuppression, nausea and vomiting, mucositis or diarrhoea (upper five histograms, Figure 1). Severe lethargy (WHO grade 3) was seen in several patients at 9 MU IFN-α, but no dose relationship was found over the range 1.5–6 MU. There were no instances of grade 4 toxicity or of neutropenia-associated fever.

Plantar–palmar erythrodysaesthesia ('hand–foot syndrome') occurred in 11 patients, usually only after more than 2 months' treatment. Several of these patients also had mild facial rashes, conjunctivitis and alopecia. Pyridoxine 50 mg t.d.s. provided partial relief from the dermatological side-effects. Other toxicities were extremely mild. One patient, at the 3 MU IFN-α level, developed mild ataxia during the FUra infusion and for 24 h afterwards, which recurred during the subsequent cycles. No cardiotoxicity was noted.

### *Anti-tumour responses*

No complete responses were seen. Among the 29 assessable patients, objective partial responses were seen in 7 of the 14 with colorectal cancer, three of the six with adenocarcinoma of unknown primary site, one of four with gastric carcinoma, none of one with pancreatic cancer and one of three with neuroendocrine tumours. All patients have subsequently relapsed; median response duration was 51 weeks (range 14–98 weeks). Two other patients had radiological responses not amounting to WHO PR, but accompanied by resolution of symptoms, signs and tumour markers ('MR'), lasting for 30 and 54 weeks. Three patients had stable disease (SD) for 22, 29 and 44 weeks. Eleven patients had progressive disease (PD) on treatment. The breakdown of response by IFN-α level is given in Table II: responses occurred at all IFN-α dose levels.

### *Survival*

After exclusion of patients treated adjuvantly and those with neuroendocrine tumours, the median survival for the 27 patients with advanced adenocarcinomas was 9 months, with 33% of patients surviving >18 months. Survival was longer for those with colorectal primaries (median survival 10.5 months) than other adenocarcinomas (median surivival 6.5 months). For comparison, in our previous study of FUra/LV without IFN-α (Johnson *et al.*, 1991), median survival was 7 months for colorectal cancer patients and 6 months for those with other adenocarcinomas.

**Figure 1** WHO toxicity scores (□, 1; ▨, 2; ■, 3) expressed as a proportion of treatment cycles administered. The bottom histogram shows, for comparison, toxicity data expressed in the same way from a previous study, in the same hospital, of 64 patients with a similar distribution of tumour types, treated using the same regimen of 5-FU and LV, without IFN-α (Johnson *et al.*, 1991).

### Discussion

The demonstration of cytotoxic synergy in preclinical models is no guarantee of a beneficial interaction in the clinic. Several issues are difficult or impossible to assess in the laboratory: exposure to one or both agents may be restricted in solid tumours; unforeseen interactions may occur in the complex biological milieu; the independent side-effects of the two agents may add up to unacceptable overall toxicity. Most importantly of all, the cytotoxic interaction observed in

**Table II** Anti-tumour responses

| *IFN-α dose level* | *PR* | *MR* | *SD* | *PD* | *NA* |
|---|---|---|---|---|---|
| 1.5 | 1 | 1 | | 2 | |
| 3.0 | 1 | | | 3 | |
| 4.5 | 1 | 1 | 2 | | |
| 6.0 | 6 | | ($1^a$) | 2 | $3^b$ |
| 9.0 | 3 | | 1 | 4 | |
| Total | 12 | 2 | 3 | 11 | 3 |

[a]One patient moved away with SD after six cycles to continue treatment elsewhere, but was never adequately reassessed. Died at 33 weeks. [b]Two treated adjuvantly; one treated with unmeasurable disease.

tumour cell lines may turn out to be just as active against key host target tissues, such as haematological or mucosal progenitors. IFN/cytotoxic drug interactions present particular difficulties to investigators since the species restriction of interferons confounds the assessment of therapeutic index in animal models.

Clinical studies of the FUra/LV/IFN-α combination must therefore address a number of separate questions:

(a) Does IFN-α potentiate the anti-tumour activity of FUra (or FUra/LV)?
(b) Does IFN-α also potentiate toxicity towards those host tissues that limit the FUra dose?
(c) If the answers to (a) and (b) are both 'yes', what is the ratio of these effects – i.e. does IFN-α improve the *therapeutic index*?
(d) Does IFN-α introduce additional, independent toxicity which limits the clinical usefulness of the combination (for example by offsetting the palliative benefit in patients with advanced disease)?

This study has established that IFN-α2a, at a dose of 6 MU on alternate days, may be added to the 'de Gramont' regimen of FUra and LV. IFN-α carries well-known and variable subjective side-effects such as lethargy and malaise, so that even at this recommended dose a proportion of patients will require dose reduction if quality of life is to be preserved. However, no correlation was found between IFN-α dose and FUra-associated toxicities in this phase I study, and only minor differences were found with historical toxicity data for FUra/LV alone. Thus we have found no evidence that IFN-α, at this dose, increases the toxicity of FUra and LV to those tissues that limit their dose.

A non-randomised study is not able to determine whether IFN-α has increased the anti-tumour effect; however, retrospective comparison may be made with a similar population of patients, treated with the same regimen of FUra/LV (without IFN-α) in our hospital, immediately prior to initiating this study. In that study, objective responses occurred in 10/33 assessable patients with colorectal cancer and 5/17 of the other adenocarcinoma patients (Johnson *et al.*, 1991). By comparison, in the current study, combining all IFN-α dose levels, responses occurred in 7/14 patients with colorectal cancer and 4/11 other adenocarcinoma patients.

Another important issue in the clinic is pharmacokinetic interaction: some studies (but not all) have suggested that, in certain schedules, IFN-α reduces the plasma clearance of FUra (reviewed in Seymour *et al.*, 1994). This has led to the suggestion that IFN-α may be acting, at least in part, as a toxic and expensive alternative to increasing the dose of FUra. However, the pharmacokinetic interaction is well established only for doses of IFN-α around 10 MU per day or higher (Grem *et al.*, 1991). In a separate report from this unit, no effect was found of IFN-α at 6 MU alternate days upon either the bolus or the infusional phases of FUra kinetics in the regimen used in this study (Seymour *et al.*, 1994).

Our results are consistent with those of other phase I trials of the combination. Grem *et al.* (1991) and Bukowski *et al.* (1992) both added daily IFN-α2a to a 5 day bolus FUra/LV regimen. The MTD of IFN-α was determined as 5 MU $m^{-2}$ $day^{-1}$ and 4 MU $m^{-2}$ $day^{-1}$ respectively. This regimen has subsequently shown promising activity in a phase II trial (Grem *et al.*, 1993). Punt *et al.* (1992) added alternate-day IFN-α2b to a 48 h infusional FUra regimen. In all of these studies, enhancement of FUra-associated toxicity (mucositis, diarrhoea, myelosuppression) was seen only at total IFN-α doses above 8–10 MU per day, the same dose range apparently required for pharmacokinetic interaction. In another trial, no enhancement of FUra/LV side-effects was detected with the addition of IFN-α2a even at 18 MU per day (Sinnige *et al.*, 1993).

Improvements in FUra therapy, even if modest, are of great clinical importance thanks to the unique status of this drug in the treatment of common cancers. IFN-α has shown clear evidence of interaction with FUra/LV in the laboratory; this study has shown that it may be added to FUra/LV in the clinic with little or no potentiation of FUra-associated toxicity, but with the addition of the usual IFN-α toxicities. Promising activity has been observed, in terms of objective tumour response rate and survival.

However, the results of this and the other phase I and II studies mentioned do not suggest that IFN-α has a very dramatic effect, and it is most unlikely that further non-randomised studies will answer the question of whether IFN-α's activity translates from the laboratory to the clinic. A prospective randomised trial is required both to determine whether IFN-α increases the anti-tumour efficacy of FUra/LV and to quantify the effects of IFN-α on the quality of life of this patient population. To this end, a multicentre randomised phase II study has just been completed in the UK, in 260 patients with advanced colorectal cancer (Medical Research Council trial CR04). The control arm is the FUra/LV regimen described in this paper (at 400 mg $m^{-2}$ FUra, bolus + infusion day 1 + 2), with patients randomised to receive, in addition, IFN-α2a 6 MU on alternate days throughout. Careful assessment is being made of anti-tumour activity in terms of objective response and survival, but also of treatment-associated toxicity and overall palliative benefit, using patient-assessed quality of life. This trial will be crucial in determining whether IFN-α has anything useful to add to the clinical activity of FUra/LV in patients with colorectal cancer.

### References

ACCMP (ADVANCED COLORECTAL CANCER META-ANALYSIS PROJECT) (1992). Modulation of fluorouracil by leucovorin in patients with advanced colorectal cancer: evidence in terms of response rate. *J. Clin. Oncol.*, **10**, 896–903.

BUKOWSKI, R.M., INOSHITA, G., YALAVARTHI, P., MURTHY, S., GIBSON, V., BUDD, B.T., SERGI, J.S., BAUER, L. & PRESTIFILIPPO, J. (1992). A phase I trial of 5-fluorouracil, folinic acid, and alpha-2a-interferon in patients with metastatic colorectal carcinoma. *Cancer*, **69**, 889–892.

CHU, E., ZINN, S., BOARMAN, D. & ALLEGRA, C.J. (1990). Interaction of γ interferon and 5-fluorouracil in H630 human colon carcinoma cell line. *Cancer Res.*, **50**, 5834–5840.

DE GRAMONT, A., KRULIK, M., CADY, J., LAGADEC, B., MAISANI, J., LOISEAU, J., GRANGE, J.-D., GONZALEZ-CANALI, G., DEMUYNCK, B., LOUVET, C., SEROKA, J., DRAY, C. & DEBRAY, J. (1988). High-dose folinic acid and 5-fluorouracil bolus and continuous infusion in advanced colorectal cancer. *Eur. J. Clin. Oncol.*, **24**, 1499–1503.

ELIAS, L. & CRISSMAN, H.A. (1988). Interferon effects upon the adenocarcinoma 38 and HL-60 cell lines: antiproliferative responses and synergistic interactions with halogenated pyrimidine antimetabolites. *Cancer Res.*, **48**, 4868–4873.

ELIAS, L. & SANDOVAL, J.M. (1989). Interferon effects upon fluorouracil metabolism by HL-60 cells. *Biochem. Biophys. Res. Commun.*, **163**, 867-874.

GREM, J.L., MCATEE, N., MURPHY, R.F., BALIS, F.M., STEINBERG, S.M., HAMILTON, J.M., SORENSEN, M., SARTOR, O., KRAMER, B.S., GOLDSTEIN, L.J., GAY, L.M., KAUBO, K.M., GOLDSPIEL, B. & ALLEGRA, C.J. (1991). A pilot study of interferon α2a in combination with fluorouracil plus high-dose leucovorin in metastatic gastrointestinal carcinoma. *J. Clin. Oncol.*, **9**, 1811-1820.

GREM, J.L., JORDAN, E., ROBSON, M.E., HAMILTON, J.M., STEINBERG, S.M., ARBUCK, S.G., BEVERIDGE, R.A., KALES, A.N., MILLER, J.A., WEISS, R.B., MCATEE, N., CHEN, A., GOLDSPIEL, B., SOVER, E. & ALLEGRA, C.J. (1993). Phase II study of fluorourcil, leucovorin and interferon-α2a in metastatic colorectal carcinoma. *J. Clin. Oncol.*, **11**, 1737-1745.

HOUGHTON, J.A., ADKINS, D.A., RAHMAN, A. & HOUGHTON, P.J. (1991). Interaction between 5-fluorouracil, [6RS] leucovorin, and recombinant human interferon-α2a in cultured colon adenocarcinoma cells. *Cancer Commun.*, **3**, 225-231.

HOUGHTON, J.A., MORTON, C.L., ADKINS, D.A. & RAHMAN, A. (1993). Locus of the interaction among 5-fluorouracil, leucovorin, and interferon-α2a in colon carcinoma cells. *Cancer Res.*, **53**, 4243-4250.

JOHNSON, P.M.W., THOMPSON, P.I., SEYMOUR, M.T., DEASY, N.P., THURAISINGHAM, R.C., SLEVIN, M.L. & WRIGLEY, P.F.M. (1991). A less toxic regimen of 5-fluorouracil and high dose folinic acid for gastrointestinal adenocarcinomas. *Br. J. Cancer*, **64**, 603-605.

KEMENY, N., YOUNES, A., SEITER, K., KELSEN, D., SAMMARCO, P., ADAMS, L., DERBY, S., MURRAY, P. & HOUSTON, C. (1990). Interferon-α2a and 5-fluorouracil for advanced colorectal carcinoma. Assessment of activity and toxicity. *Cancer*, **66**, 2470-2475.

LOKICH, J.J., AHLGREN, J.D., GULLO, J.J., PHILIPS, J.A. & FRYER, J.G. (1989). A prospective randomized comparison of continuous infusion fluorouracil with a conventional bolus schedule in metastatic colorectal carcinoma: a Mid-Atlantic Oncology Program study. *J. Clin. Oncol.*, **7**, 425-432.

NEEFE, J.R. & GLASS, J. (1991). Abrogation of interferon-induced resistance to interferon-activated major histocompatibility complex-unrestricted killers by treatment of a melanoma cell line with 5-fluorouracil. *Cancer Res.*, **51**, 3159-3163.

PETRELLI, N., DOUGLASS, H.O., HERRERA, L., RUSSELL, D., STABLEIN, D.M., BRUKNER, H.W., MAYER, R.J., SCHINELLA, R., GREEN, M.D., MUGGIA, F.M., MEGIBOW, A., GREENWALD, E.S., BUKOWSKI, R.M., HARRIS, J., LEVIN, B., GAYNOR, E., LOUTFI, A., KALSER, M.H., BARKIN, J.S., BENEDETTO, P., WOOLEY, P.V., NAUTA, R., WEAVER, D.W. & LEICHMAN, L.P. (1989). The modulation of fluorouracil with leucovorin in metastatic colorectal carcinoma: a prospective randomized phase III trial. *J. Clin. Oncol.*, **7**, 1419-1426.

POON, M.A., O'CONNELL, M.J., WIEAND, H.S., KROOK, J.E., GERSTNER, J.B., TSCHETTER, L.K., LEVITT, R., KARDINAL, C.G. & MAILLIARD, J.A. (1991). Biochemical modulation of fluorouracil with leucovorin: confirmatory evidence of improved therapeutic efficacy in advanced colorectal cancer. *J. Clin. Oncol.*, **9**, 1967-1972.

PUNT, C.J.A., DE MULDER, P.H.M., BURGHOUTS, J.T.M. & WAGENER, D.J.T. (1992). Alpha-interferon in combination with 5-fluorouracil and leucovorin in metastatic colorectal cancer: a phase I study. *Cancer Chemother. Pharmacol.*, **29**, 326-328.

SCHWARTZ, E.L., HOFFMAN, M., O'CONNOR, C.J. & WADLER, S. (1992). Stimulation of 5-fluorouracil metabolic activation by interferon-α in human colon carcinoma cells. *Biochem. Biophys. Res. Commun.*, **182**, 1232-1239.

SEYMOUR, M.T., DOBSON, N., CLEMENS, M.J. & SLEVIN, M.L. (1992*a*). 5-Fluorouracil interferon-α synergy: is regulation of expression of thymidylate synthase the key? *Proc. Annu. Meet. Am. Assoc. Cancer Res.*, **33**, 545.

SEYMOUR, M.T., MCSHEEHY, P.M.J., RODRIGUES, L. & GRIFFITHS, J.R. (1992*b*). Modulation by interferon-α of 5-fluorouracil kinetics in human colon tumour HT29 grown in nude mice: a $^{19}$F-MRS study. *Soc. Magnetic Resonance Med.*, **11**, 3514.

SEYMOUR, M.T., PATEL, N., JOHNSTON, A., JOEL, S.P. & SLEVIN, M.L. (1994). Lack of effect of interferon-α2a upon fluorouracil pharmacokinetics. *Br. J. Cancer*, **70**, 000-000.

SHAH, A., MACDONALD, W., GOLDIE, J., GUDAUSKAS, G. & BRISEBOIS, B. (1985). 5-Fluorouracil infusion in advanced colorectal cancer: a comparison of three dose schedules. *Cancer Treat. Rep.*, **69**, 739-742.

SINNIGE, H.A.M., BUTER, J., DE VRIES, E.G.E., UGES, D.R.A., ROENHORST, H.W., VERSCHUEREN, R.C.J., SLEIJFER, D.T., WILLEMSE, P.H.B. & MULDER, N.H. (1993). Phase I-II study of the addition of α2a interferon to 5-fluorouracil leucovorin. Pharmacokinetic interaction of α2a interferon and leucovorin. *Eur. J. Cancer*, **29A**, 1715-1720.

WADLER, S. & SCHWARTZ, E.L. (1990). Antineoplastic activity of the combination of interferon and cytotoxic agents against experimental and human malignancies: a review. *Cancer Res.*, **50**, 3473-3486.

WEINERMAN, B., SHAH, A., FIELDS, A., CRIPPS, I.C., WILSON, K., MCCORMICK, R., TEMPLE, W., MAROUN, J., BOGUES, W. & PATER, J. (1992). Systemic infusion versus bolus chemotherapy with 5-fluorouracil in measurable metastatic colorectal cancer. *Am. J. Clin. Oncol.*, **15**, 518-523.

WHO (WORLD HEALTH ORGANIZATION) (1979). *Handbook for Reporting Results of Cancer Treatment*. World Health Organisation Offset Publication No. 48. WHO: Geneva.

YEE, L.K., ALLEGRA, C.J., STEINBERG, S.M. & GREM, J.L. (1992). Decreased catabolism of fluorouracil in peripheral blood mononuclear cells during combination chemotherapy with fluorouracil, leucovorin and interferon-α2a. *J. Natl Cancer Inst.*, **84**, 1820-1825.